# Impact of Adsorption on Gas Transport in Nanopores

**DOI:** 10.1038/srep23629

**Published:** 2016-03-29

**Authors:** Tianhao Wu, Dongxiao Zhang

**Affiliations:** 1 Department of Energy and Resources Engineering, College of Engineering, Peking University, Beijing 100871, China; 2 ERE & BIC-ESAT, College of Engineering, Peking University, Beijing 100871, China

## Abstract

Given the complex nature of the interaction between gas and solid atoms, the development of nanoscale science and technology has engendered a need for further understanding of gas transport behavior through nanopores and more tractable models for large-scale simulations. In the present paper, we utilize molecular dynamic simulations to demonstrate the behavior of gas flow under the influence of adsorption in nano-channels consisting of illite and graphene, respectively. The results indicate that velocity oscillation exists along the cross-section of the nano-channel, and the total mass flow could be either enhanced or reduced depending on variations in adsorption under different conditions. The mechanisms can be explained by the extra average perturbation stress arising from density oscillation via the novel perturbation model for micro-scale simulation, and approximated via the novel dual-region model for macro-scale simulation, which leads to a more accurate permeability correction model for industrial applications than is currently available.

The transport mechanism of gas through nanopores is a fundamental concern in many fields, such as nanofluidics, fluid mechanics, material science, chemical engineering, and petroleum engineering, which has attracted considerable attention. For example, with the successful development of unconventional oil and gas^1^,^2^,^3^, obtaining a better understanding of the transport mechanisms in the nanopores of tight/shale matrix is important for predicting long-term gas production; on the other hand, the discovery of the rapid transport of gases in carbon nanotubes and graphene membranes^4^,^5^,^6^ means that these materials have the potential to function as high-efficiency and low-cost materials for hydrogen or methane storage. All of these applications involve the movement of gas molecules through highly confined space where the gas molecules are strongly affected by gas–solid interaction in addition to purely gas–gas interaction^7^,^8^. Because the strong density oscillation of fluid atoms near the fluid–solid interface is a universal phenomenon^9^, the gas–solid interaction can be described with the help of the concept of physical adsorption^10^,^11^,^12^, which is a well-developed field in physical chemistry. The effects of adsorption cannot be neglected, since the surface-to-volume ratio is very high, and the critical dimension is comparable to the thickness of the adsorption layer in a nanoscale system^9^. Therefore, the mechanisms of gas transport under the influence of adsorption in nanopores need to be understood in detail.

Research on gas flow in confined spaces has a long history, dating back to the works of Maxwell^13^ and Knudsen^14^. They presented the phenomenon of gas slippage near a solid wall in the context of the gas–solid collision, and the models were subsequently refined by others^15^. Then, Klinkenberg addressed the enhanced gas flow in a porous medium and its effect on permeability^16^. As the adsorption phase contributes to transport of gases to a great extent in some cases, surface diffusion was introduced to explain the mass flow in excess to that predicted by Knudsen’s model, and on this bases more models were proposed and improved^17^,^18^,^19^,^20^,^21^. On the other hand, the adsorbing capacity were often modeled by various adsorption isotherms, such as the Langmuir model for monolayer adsorption^22^ and the Brunauer-Emmett-Teller (BET) model for multilayer adsorption^23^. In general, the above models are expressed in the diffusional framework with a concentration dependent diffusivity. However, another way of investigating the transport mechanisms is based on the Navier-Stokes (N-S) equation framework with idealized models, such as the channel flow and the tube flow^24^. The flow under nanoscale exhibits non-continuum effects^25^, and its flow regimes can be classified as continuum flow, slip flow, transition flow, and free molecular flow^26^ by Knudsen number (*Kn* = *λ*/*l*, where *λ* is the molecular mean free path, and *l* is the characteristic length). As the characteristic length approaches the order of the molecular mean free path, the *Kn* of the gas flow in nanopores is in a similar range to that of the rarefied gas flow with large *λ* under low pressure. Then, the law of similarity can be applied, which is in the slip flow or transition flow regime^24^ with a low Reynolds number and a low Mach number. Further, the continuum model with corrections, such as the N-S equation, has been utilized to describe the gas flow in nanopores, which were proved through direct-simulation Monte Carlo (DSMC) and linearized Boltzmann equations^24^. After that, a variety of slip boundary conditions^24^,^27^,^28^,^29^, such as the extended N-S equation^30^ and empirical velocity profiles^25^,^31^,^32^ were developed and widely applied. In general, because of the weak contribution of adsorption in rarefied gas flow under ultra-low-pressure conditions, the adsorption effect is often neglected in these models. However, high pressure, corresponding to dense gas, is also very common in industrial applications, and the impact of adsorption may be significant on the gas transport process. In addition, transport behaviors are often described as a variety of mechanisms, such as Knudsen diffusion, slip flow, viscous flow, and/or surface diffusion, and the dominant mechanisms may vary under different conditions. Subsequently, some phenomenological models^33^,^34^,^35^,^36^ in a superposition form of the above mechanisms were proposed, and were utilized to interpret experimental results. However, the rationality of some phenomenological models is under debate, due to their insufficiency to address the coupled effect.

With the development of atomistic simulation methods and computing capacity, the Molecular Dynamics (MD) simulation has become a powerful tool for investigating the micro-scale mechanisms. Various MD simulation techniques, such as equilibrium molecular dynamics (EMD), non-equilibrium molecular dynamics (NEMD), and dual control volume grand canonical molecular dynamics (DCV-GCMD), were designed with different strengths and applied to verify theoretical models and discover novel phenomena^37^,^38^,^39^. For example, the large slip lengths in graphite slit-like pores and carbon nanotubes were presented, respectively, and an interfacial friction model was proposed, which indicated that the behavior was influenced by the surface corrugation and the fluid–wall interaction strength^40^,^41^; another friction-based model demonstrated that the combination of viscous flow and momentum exchange at the pore wall governs the transport^42^; the oscillator model, on the other hand, offered an exact theory for low density gas flow^8^; and sub-continuum mass transport of condensed hydrocarbon in disordered porous carbon, with pore sizes ranging from 0.3 to 1.3 nm, was investigated^43^. Overall, various phenomena have been discovered and modeled independently, however, the understanding of the mechanisms of gas transport coupled with adsorption in nanopores remains limited, and a tractable numerical model for large-scale simulation under an extensive range of pore size and pressure has yet to be proposed.

In the current paper, we present a theoretical study of the impact of adsorption on gas transport in nanopores, which aims at analyzing the coupled behavior in detail and proposing numerical models for the micro-scale phenomena description and macro-scale flow simulation. First, we perform MD simulations to show the special mass flow properties in the nano-channel made of illite and graphene, respectively. To account for the velocity behavior under the strongly heterogeneous gas density distribution along the cross-section of the channel, we propose a perturbation model, which decomposes the stress term into viscous stress and average perturbative stress. As the monolayer adsorption assumption remains valid for the majority of gas transport cases, we present a dual-region model in order to approximate the mass flow and demonstrate its application of permeability correction. Meanwhile, other classical models and frequently utilized models are compared within this paper.

## Results

### MD simulations of gas transport in nano-channels

We constructed the nano-channel (slit-like pore) structures with illite^44^ and graphene, respectively. Illite represents a common layered clay mineral with significant adsorption in the shale matrix. Graphene, on the other hand, represents the advanced material for various applications including hydrogen storage^45^ and methane storage^46^. In the case of illite, the silicon-oxygen layer was assigned as the pore surface, which can represent many frequently utilized solid structures. The center of the top layer’s atoms was set as the position of the solid wall. The effective pore width was smaller than the assigned value and may change according to the atoms’ properties. Two kinds of NEMD simulations were conducted for isothermal methane flow as a simple fluid at room temperature (*T* = 298 K). One was based on the reflecting particle method (RPM), which is easy to apply and the driving mechanism of which is close the actual pressure-driven flow^38^. As the pressure gradient in the highly confined space could not be well-defined or monitored, the other one, the external force method (EFM), was applied in the next section in order to accurately determine the pressure gradient. Supplementary Fig. S1 presents the comparison of RPM and EFM for this case. Additional details pertaining to the MD simulation methods and settings can be found in Supplementary Methods.

**Figure 1:**
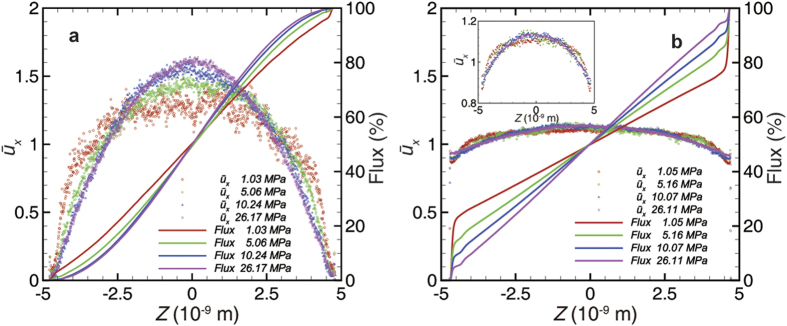
Transport behavior under various pressure conditions in a 10-nm-wide channel from MD simulations. Velocity (dot) and accumulative mass flux (line) in the illitic channel (**a**) and the graphene channel (**b**) along the cross-section; inset: velocity profile after zooming in on the y-axis in graphene channel cases. The corresponding gas atoms number density distribution is shown in Supplementary Fig. S2. The pressure is the average value in the central free space with uniform density distribution. The velocity is normalized by the average velocity across the channel.

Figure 1 demonstrates the velocity profile and the accumulative mass flow across the 10-nm-wide channel under various pressure conditions. In the illite cases, the velocity profile can be divided into three regions. The first region is adjacent to the solid atoms where the gas atoms are strongly repulsed by the wall and no atoms or velocity appears. Therefore, this region can be eliminated by defining the effective width. The second region is around the adsorbed region where complex phenomena are present. The slip velocity is very small, and vanishes in the high-pressure case. The velocity gradient is smaller around the potential well, where a density peak exists, than that in the other regions. The third region is the inner region, the transport behavior of which mainly follows the classical parabolic type but with different curvatures caused by the rarefaction effect in the nanopores^24^. As the pressure decreases, the velocity profile transforms from the parabolic type to the plug type, due to the viscosity reduction as the Knudsen number increases. Additionally, a velocity inflection point always exists between the inner region and the adsorbed region. On the whole, due to the small velocity at the boundary, the adsorbed region contributes very little to the total mass flow, especially in the high-pressure case, which is consistent with the macroscopic behavior of shale gas production^47^. However, although the graphene cases also have three regions, a significant phenomenon arises. A large slip velocity, principally due to the ultra-smooth surface of the graphene, appears in the adsorbed region. Since the density peak is in the adsorbed region and the difference in velocity between the adsorbed region and the inner region is minute, the adsorbed region contributes substantial mass flux, even exceeding 40% in the relatively low-pressure case. This nice property has been explored in order to determine its potential in fast gas storage, the diffusivity of which was shown to be several orders of magnitude higher than that of normal nano-porous media^4^. The relative contribution of the adsorbed region decreases as the pressure increases, because the density ratio between the adsorbed region and the inner region decreases. Taken as a whole, the velocity profile fits a flat type, but with a similar trend under various pressures to that of the illite case (Fig. 1b inner plot).

### Perturbation Model

The molecular transport at the micro-scale can be easily decomposed into the streaming velocity and the thermal motion velocity or speed^48^,^49^, namely, 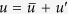. When we monitor the average streaming velocity in a specific bin, it is implicitly assumed that the velocity is a density-weighted average velocity, because the average density is also a statistical value and the heterogeneous density distribution exists in a nanopore. This definition can be expressed as 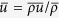, where *u* is the velocity of the molecule, *ρ* is the density, and 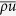 and 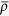 denote the long-time average of momentum and of density in a specific bin, respectively. We use 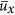 in the figures to represent the x-component of the average streaming velocity. If we take a long-time accumulation for the thermal velocity, the value should be zero. We can, therefore, treat the thermal velocity as a perturbative velocity. If we assume that the continuum theory is valid in this condition from a statistical perspective, it is reasonable to apply the Navier-Stokes equation framework^30^. In analogy to the turbulence theory, which often employs molecular transport as an analogy in return^50^, the two theoretical systems maintain the same decomposition scheme. Therefore, we propose a perturbation model to describe the flow behavior in nanopores:


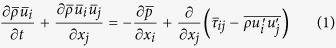


where *t* represents time; 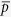 stands for the average pressure; 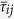 signifies the stress term; 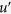 is the perturbation velocity; 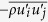 is the extra average perturbation stress term; and *i*, *j* denotes the axis-direction. This model retains the same form as the compressible turbulence flow based on the mass-weighted average concept^50^. In order to determine a physical expression of the perturbation term, we can apply a simplified kinetic theory model for approximation. Then, if we define 
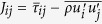
 for the stress term, the momentum transport across the unit area can be denoted as:


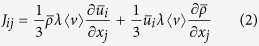


where 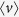 is the thermal motion speed. The derivation is given in Supplementary Methods. The first term can be expressed as the viscosity term in the relationship of 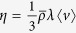, where the viscosity *η* can be assumed to be independent of the density in gas flows^49^ and kept constant under certain temperature. For a Newtonian fluid, the stress due to viscosity can be expressed as:


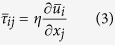


Additionally, we can assume that:


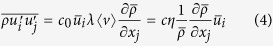


where *c*_0_ and *c* are scale coefficients to be fitted. Then, equation (4) indicates that the velocity oscillation may result from the extra average perturbation stress due to density oscillation.

According to the configuration of the model in the MD simulations, the equations can be reduced to:


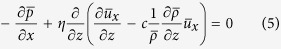


Equation (5) is solved via the finite difference method with the no-slip boundary condition at the wall atoms’ center position and the symmetric boundary at the center of the pore. The density profile is the most important input information.

**Figure 2:**
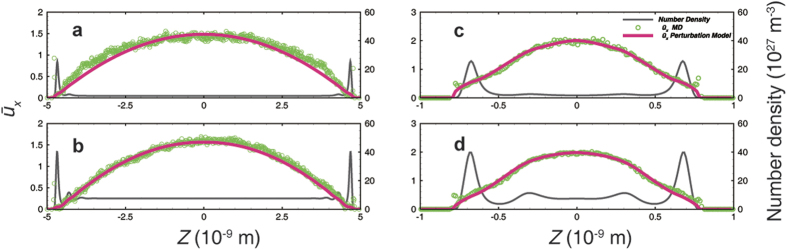
Velocity fitting results from the perturbation model in the illitic channel. (**a**) Low-pressure case (5.06 MPa) in the 10-nm-wide channel. (**b**) High-pressure case (26.17 MPa) in the 10-nm-wide channel. (**c**) Low-pressure case (7.44 MPa) in the 2-nm-wide channel. (**d**) High-pressure case (27.74 MPa) in the 2-nm-wide channel. The pressure is the average value in the central free space with a uniform density distribution in (**a,b**). The pressure cannot be well defined in a 2-nm-wide channel, therefore, the pressures in (**c,d**) are representative values, just for comparison, from the average density via the Peng-Robinson equation of state. The velocity is normalized by the average velocity across the entire channel from the MD simulation. The post-processing methods for pressure and velocity are also applied in all other figures presented in this work.

**Figure 3:**
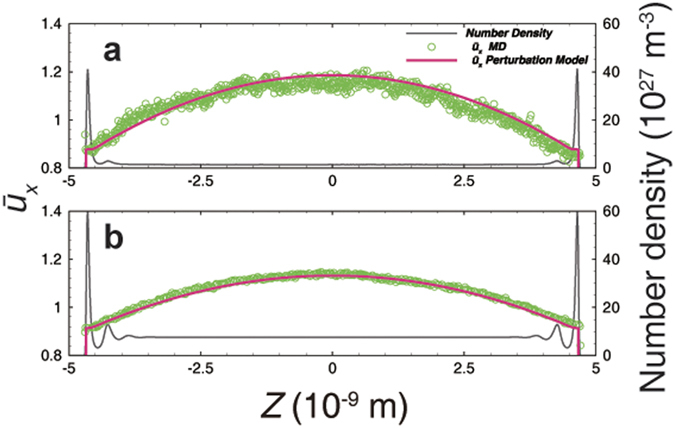
Velocity fitting results from the perturbation model in a 10-nm-wide graphene channel. (**a**) Low-pressure case (5.16 MPa). (**b**) High-pressure case (26.11 MPa).

**Figure 4:**
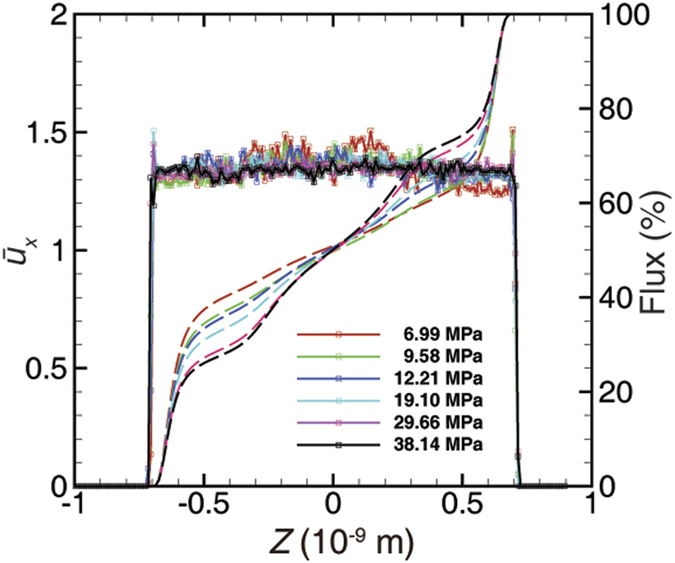
Transport behavior in a 2-nm-wide graphene channel under various pressure conditions. Velocity (line-dot) agrees with the flat type, and the oscillation is covered by noise; the accumulative mass flux curve (dashed line) is only affected by the density distribution.

To verify the perturbation model, MD simulations were performed by imposing the pressure gradient through external force. Figure 2 demonstrates the velocity profile results from the illite cases with various channel widths. The perturbation model can thoroughly capture the features of velocity profiles, accurately describing the inflection point which is more significant in the 2 nm cases. The scale coefficient *c* is 0.0370, 0.0105, 0.0175, and 0.0194 in the cases demonstrated in Fig. 2a–d, respectively. The viscosity was calculated with the LBC method and corrected with the rarefaction effect^24^. Because of the smooth surface of graphene, the gas density profile always maintains a very sharp peak adjacent to the wall (see Supplementary Fig. S2), which often leads to numerical instability at the boundary. However, if an average velocity in the adsorbed region near the wall is assigned to the boundary, the perturbation model can also capture the velocity properties in both the adsorbed region and the inner region. The scale coefficient *c* is 2.4 × 10^−4^ and 5.8 × 10^−4^ in the cases demonstrated in Fig. 3a,b, respectively. The 2 nm graphene cases present flat velocity profiles (Fig. 4), because the slip velocity is very large and the effects of viscosity and density oscillation are covered by noise. In addition, the mass flux contribution corresponds to the density profile. Therefore, it is not necessary to fit the model in this case.

It should also be noted that the perturbation model offers an explanation of the slippage mechanism. The slip velocity or the small slip region near the wall can be described well by this model in the illite case (see Fig. 2c,d). In the graphene case, the large slip velocity may be related to the large density gradient near the wall, which is much larger than in the illite case (see Supplementary Fig. S2). Although no wall potential term appears in this model, the density profile is an effective indirect parameter containing not only the effect of the solid wall but also the interaction between the gas–gas molecules. In particular, this model avoids the problem encountered in the continuum method in which the solid wall’s effect is unmanageable. Furthermore, this model makes it possible to apply density profile information obtained from other methods, e.g., grand canonical molecular dynamics (GCMC) and density function theory (DFT), to gas flow simulation.

### Dual-Region Model

The perturbation model can describe the velocity profile very well at the micro-scale and offer a basis for gaining insights into and even explaining the mechanisms. However, applying the perturbation model in macro-scale simulations is challenging because it is difficult to obtain the density distribution within each of the pores for the macro-scale pore network model. From the MD results and the perturbation model, we found that the behavior of the velocity profile differs significantly between the adsorbed region and the inner region because of the density oscillation. We can assume two surfaces, corresponding to the positions of the large density valleys: one surface, which may have slip velocity, is between the solid wall and the adsorbed region, whereas the other surface, which is virtual in nature, is between the first adsorbed layer and the inner region. Although the second density peak appeared in the high-pressure case, it simply enhanced the velocity oscillation around the surface, and no obvious oscillation presented between the second density peak and the central region. In other words, only the monolayer adsorption atoms maintained a significant effect on gas flow. Therefore, it is reasonable to propose simplified models for macro-scale simulation to approximate this flow behavior.

Here, we propose a dual-region model (D-R model) for mass flow in nanopores, based on the partition of the adsorbed region and the inner region, the flow regimes of which differ. The density distribution is assumed to be uniform within each region. Additionally, the density of the adsorbed region is easy to determine from experiments, such as the adsorption isotherm test and the specific area test. To approximate the major velocity features in each region, we sacrificed the velocity precision around the virtual surface, which we treated it as a slip velocity. The slip boundary conditions were applied for both surfaces through the respective Knudsen number and the tangential momentum accommodation coefficient (TMAC), which may switch to the no-slip boundary under high pressure^24^. The viscosities were also corrected with *Kn* and density in each region, respectively. Therefore, the approximate velocity profile can be expressed as follows by taking the center of the pore as the zero point:


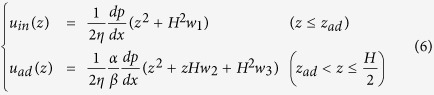


where *H* is the effective channel width; *α* and *β* are the ratios of the density and viscosity between the adsorbed region and the inner region, respectively; *w*_1_, *w*_2_ and *w*_3_ are coefficients in terms of *α*, *β*, *Kn*, TMAC, and the adsorbed region’s width (for expressions, see Supplementary Methods); the width of the adsorbed region is assigned as *γσ*, where *σ* corresponds to the distance at which the potential between the two atoms goes to zero, which can be approximately taken as the diameter of a gas atom, and *γ* is set to 0.8 for methane empirically; the subscripts *in* and *ad* denote the inner region and the adsorbed region, respectively; and *Z*_*ad*_ is the location of the virtual surface. Then, we can obtain the total mass flux as:


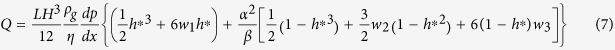


where *L* · *H* is the cross area of the channel; *ρ*_g_ is the gas density in the inner region; and h^*^ is the width ratio of the inner region and the entire channel. Other classical models, including the no-slip model and the Beskok-Karniadakis-Trimmer (BKT) model^24^,^27^ are introduced for comparison. The BKT model is widely applied for microflows and nanoflows, which are proposed based on rarefied gas flow and fail to account for adsorption. Notably, the dual-region model will reduce to the BKT model in the limiting cases, such as those in which no adsorption effect exists.

**Figure 5:**
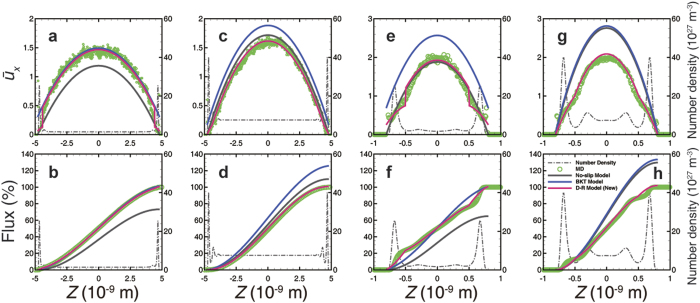
Comparisons of different models for velocity and accumulative mass flux in the illitic channel. Upper panel: velocity profile. Lower panel: accumulative mass flux. (**a,b**) 10-nm-wide, low-pressure (5.06 MPa); (**c,d**) 10-nm-width, high-pressure (26.17 MPa); (**e,f**) 2-nm-width, low-pressure (7.44 MPa); (**g,h**) 2-nm-width, high-pressure (27.74 MPa). The values of TMAC can be found in Supplementary Table S1.

Figure 5 demonstrates the comparisons with the no-slip model and the BKT model in the illite case. In case 1 (Fig. 5a,b, width: 10 nm, low-pressure), both the dual-region model and the BKT model agree with the profile very well, whereas the no-slip model underestimates both the velocity and the mass flow. In case 2 (Fig. 5c,d, width: 10 nm, high-pressure), both the BKT model and the no-slip model overestimate the velocity and the mass flow rate. The BKT model always maintains slip velocity, because the bulk *Kn* is not small enough to obtain a no-slip boundary in such a narrow channel. The no-slip model cannot correct the viscosity in the adsorbed region like the dual-region model does. In contrast, the dual-region model maintains a very small *Kn* in the adsorbed region so that the slip velocity approaches zero, and can apply viscosity correction with each region’s *Kn*. However, in case 3 (Fig. 5e,f, width: 2 nm, low-pressure), the no-slip model agrees with the velocity profile very well but underestimates the flow rate, because this model cannot account for the density difference, which has little effect in cases 1 and 2 due to the small volume fraction of the adsorbed region. Although the BKT model could fit the mass flow profile by adjusting the TMAC, this model would have non-physical velocity. Case 4 (Fig. 5g,h, width: 2 nm, high-pressure) resembles case 2 but with a more sizable deviation, since the adsorbed region maintains a larger volume fraction.

**Figure 6:**
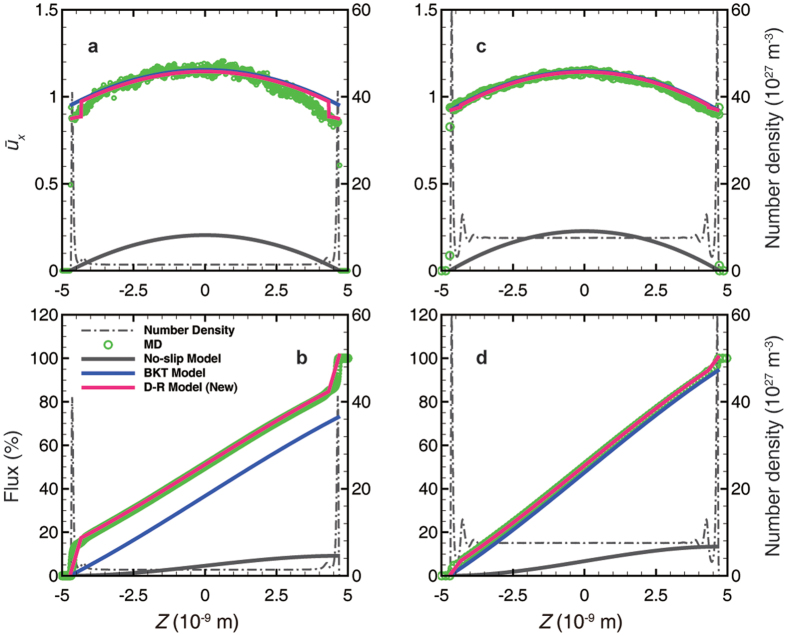
Comparisons of different models for velocity and accumulative mass flux in a 10-nm-wide graphene channel. Upper panel: velocity profile. Lower panel: accumulative mass flux. (**a,b**) Low-pressure (5.16 MPa). (**c,d**) High-pressure (26.11 MPa). The values of TMAC can be found in Supplementary Table S2.

In case 5 (Fig. 6a,b, width: 10 nm, low-pressure), because of the large slip velocity, the no-slip model significantly underestimates the velocity and the mass flow, whereas the BKT model and the dual-region model both agree very well with the MD results in the central major part. In addition, the dual-region model has an adjustment in the adsorbed region. However, the BKT model still underestimates the total mass flow because the enhanced density in the adsorbed region is not accounted for. Nevertheless, this deviation decreases, as the density ratio of the adsorbed region and inner region decreases with increasing pressure in case 6 (Fig. 6c,d, width: 10 nm, high-pressure).

Overall, the dual-region model can capture most of the features in nano-channels with the addition of three parameters (*α*, *β*, and h^*^), each of which has a definite physical meaning and can be estimated from theoretical calculations or measured from experiments. However, the classical no-slip model and the BKT model may either overestimate or underestimate the velocity and the mass flow. Although the BKT model can fit the total mass flow in some cases, this model may have a non-physical velocity. Therefore, the dual-region model offers a better approximate model for the macro-scale simulation than other models, since it accounts for the adsorption effect and the complex flow mechanisms.

### Macro-scale Permeability Correction

From the macroscopic perspective, the performance of gas transport through a porous medium is often denoted as the apparent permeability^51^, which can be expressed as the intrinsic permeability multiplied by the correction factor *f*. The intrinsic permeability is an inherent property of a porous medium, and does not depend on either fluid type or flow-regime. The BKT model can also convert to a correction factor function in terms of *Kn*, which can be expressed as^24^:


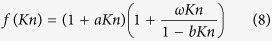


where *a* is a rarefaction coefficient for viscosity correction; *b* is a slip coefficient and often taken as −1 for channel flow and tube flow; *ω* = 6 in the channel flow and *ω* = 4 in the tube flow. For the dual-region model, the correction factor function is:


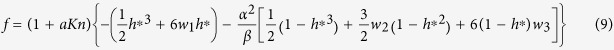


**Figure 7:**
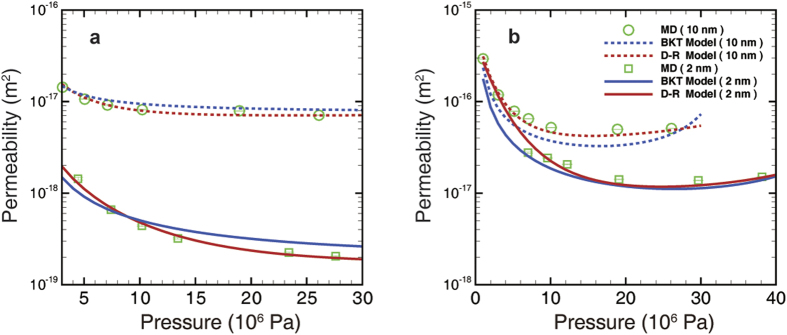
Apparent permeability of the nano-channel. (**a**) Illitic channel. (**b**) Graphene channel. Error bars of MD simulations are smaller than the symbol sizes.

In the channel flow, the intrinsic permeability can be denoted as 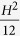 based on the Stokes’ equation. Then, the MD simulation results can be interpreted in the form of the apparent permeability. As shown in Fig. 7a,b, the dual-region model maintains a more effective performance throughout all of the cases than the BKT model. The Langmuir-type adsorption isotherms are applied as input parameters for the apparent permeability calculation (see Supplementary Fig. S3). In addition, if we convert the apparent permeability to transport diffusion coefficient form, the diffusivity of the graphene channel maintains a similar value to the (10, 10) carbon nanotube^4^ (about 1 × 10^−4^ m^2^ s^−1^).

To propose a correction factor model for industrial applications, the dual-region model is extended to the shale matrix permeability correction model, and a theoretical comparison with the BKT model is performed. The correction factor function based on the dual-region model is:


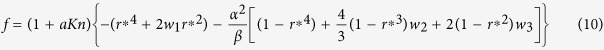


where r^*^ is the radius ratio of the inner region and the entire pore and the width of the adsorbed region is set as *γσ*.

The presented intrinsic permeability values are 10 nD, 100 nD, and 1,000 nD (1 nD = 10^−9^ D = 9.869 × 10^−22^ m^2^), and the corresponding representative pore radius is in the empirical relationship^52^ of:


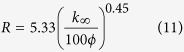


where *k*_∞_ is the intrinsic permeability and *ϕ* is the porosity. With the adsorbed region’s effect taken into consideration, the Langmuir-type adsorption isotherm is also applied, and the effect of Langmuir adsorption volume *V*_*L*_ is compared. TMAC is assigned as 1 for the cases. Other parameters are set to be equal with those of the BKT model, and the parameters are in a reasonable range for actual shale gas reservoirs^47^ (see Supplementary Table S3 and Supplementary Methods).

**Figure 8:**
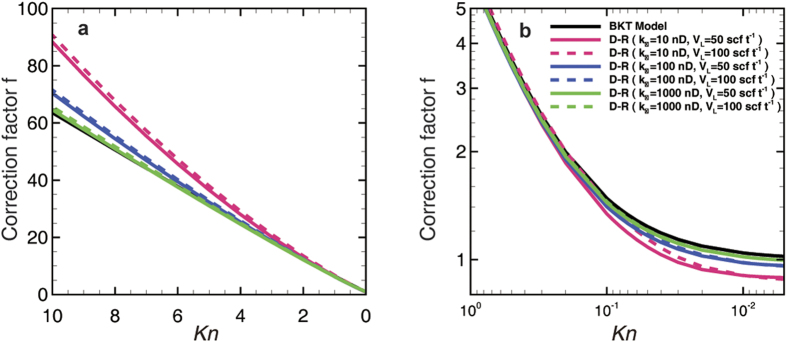
Permeability correction factor for shale matrix. (**a**) The correction factor vs. the Knudsen number. The curve of the BKT model almost coincides with that of the 1,000 nD case. (**b**) Zoom in on the plot for the low-Knudsen-number region with log-axis. Except for in the 10 nD case, the curves of the Langmuir adsorption volumes (1 scf ton^−1^ = 2.83 × 10^−5^ m^3^ kg^−1^) are almost coincident.

Figure 8a shows that the correction factor is larger than that predicted by the BKT model in the high-*Kn* regime (or transition regime) because of the extra contribution in the adsorbed region. The underestimation by the BKT model increases as the *k*_∞_ decreases, corresponding to the decrease of *R*, due to the increase of the adsorbed region’s volume proportion. On the other hand, a larger *V*_*L*_ results in a larger correction factor with the same *k*_∞_, and the difference increases with the decrease of *k*_∞_ for the same reason. In general, the BKT model underestimates the apparent permeability under relatively low-pressure conditions in ultra-tight reservoirs by neglecting the effect of adsorption.

In contrast to the high-*Kn* regime, the BKT model, as shown in Fig. 8b, will overestimate the apparent permeability in the low-*Kn* regime (i.e., the slip or viscous regime). Further, the BKT model’s deviation also increases with the decrease of *k*_∞_ (Fig. 8b), which corresponds to the effects shown in Fig. 5c,d,g,h. However, *V*_*L*_ does not have much influence in either the 100 nD cases or the 1,000 nD cases because the velocity in the adsorbed region is very small. Nevertheless, a small difference of the correction factor is presented in the 10 nD cases because the adsorbed region maintains a large enough proportion to show the minute differences in the results depending on different *V*_*L*_ values. On the whole, adsorption has an obstructive effect on the transport under relatively high-pressure conditions, the magnitude of which is related to the volume proportion of the adsorbed region accordingly. Furthermore, the correction factor from the BKT model will never be less than 1 according to equation (8), because the BKT model only considers the enhancement of slippage and the rarefaction effect, which is not the case under the high-pressure and narrow-pore conditions. Therefore, the correction factor model based on the dual-region model establishes a more accurate correction method by accounting for adsorption, which can be utilized as an up-scaled model in macroscopic modeling and simulation.

## Discussion

In the present study, we have demonstrated the complex mechanisms of gas transport under the influence of adsorption in nano-channels composed of different materials. As a novel alternative to the continuum method, the perturbation model accurately describes the microscopic phenomenon of velocity oscillation by taking into account the density distribution across the channel, which is the result of the combination of gas–solid and gas–gas interactions. Additionally, this model provides a link between the microscopic method and the macroscopic method, which can introduce the results from the non-continuum method to the continuum method, as well as constituting a potential method for large-scale high-precision simulations based on the continuum description. In reference to the mechanisms of velocity oscillation as expressed by the perturbation model, the dual-region model offers a simplified approach to larger-scale simulation in the context of contemporary computing capability. As a novel method to handle the adsorption effect, the dual-region model also offers a correction factor model for the quantitative prediction of total mass flow with performance that is superior to that of classical models. In particular, the shale apparent permeability correction case presents an up-scaled model that have potential to be employed in macroscopic modeling and simulation for industrial application. Nevertheless, this study is still limited to the gas transport of simple fluid, while generalized model involved complex fluid remains to be developed. For the up-scaled model, the systematical analysis of uncertainty, which usually comes from the heterogeneity, tortuosity, and determination of characteristic radius, is also worthy to be investigated in the future work for the industrial application. Overall, the present study offers an in-depth theoretical investigation of the mechanisms and the impact of adsorption on gas transport in nanopores from phenomena to models and their applications.

## Additional Information

**How to cite this article**: Wu, T. and Zhang, D. Impact of Adsorption on Gas Transport in Nanopores. *Sci. Rep.* **6**, 23629; doi: 10.1038/srep23629 (2016).

## Supplementary Material

Supplementary Information
