# Supplementary material for: Impact of Adsorption on Gas Transport in Nanopores
Source: Sci Rep. 2016 Mar 29;6:23629. doi: 10.1038/srep23629 (PMC4810319; doi:10.1038/srep23629)
Supplement: Supplementary Information [file srep23629-s1.pdf]

Title: Impact of Adsorption on Gas Transport in Nanopores

Tianhao Wu

Department of Energy and Resources Engineering, College of Engineering, Peking University, Beijing 100871, China.

wutianhao@pku.edu.cn

Dongxiao Zhang* (Corresponding author)

ERE & BIC-ESAT, College of Engineering, Peking University, Beijing 100871, China.

dxz@pku.edu.cn

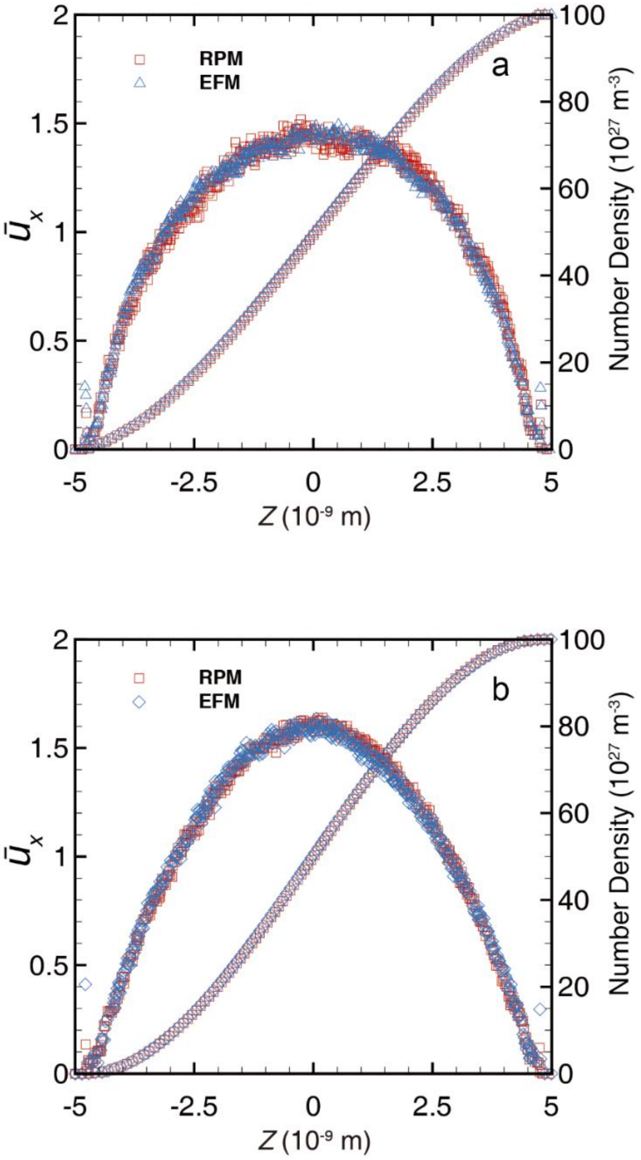

Supplementary Figure S1 | Comparisons between the reflecting particle method (RPM) and the external force method (EFM) in a 10-nm-wide illitic channel. (a) Low-pressure (5.06 MPa). (b) High-pressure (26.17 MPa). We monitored the pressure gradient in the RPM case; then, we calculated the external force to obtain the equivalent pressure gradient in the other case for comparison.

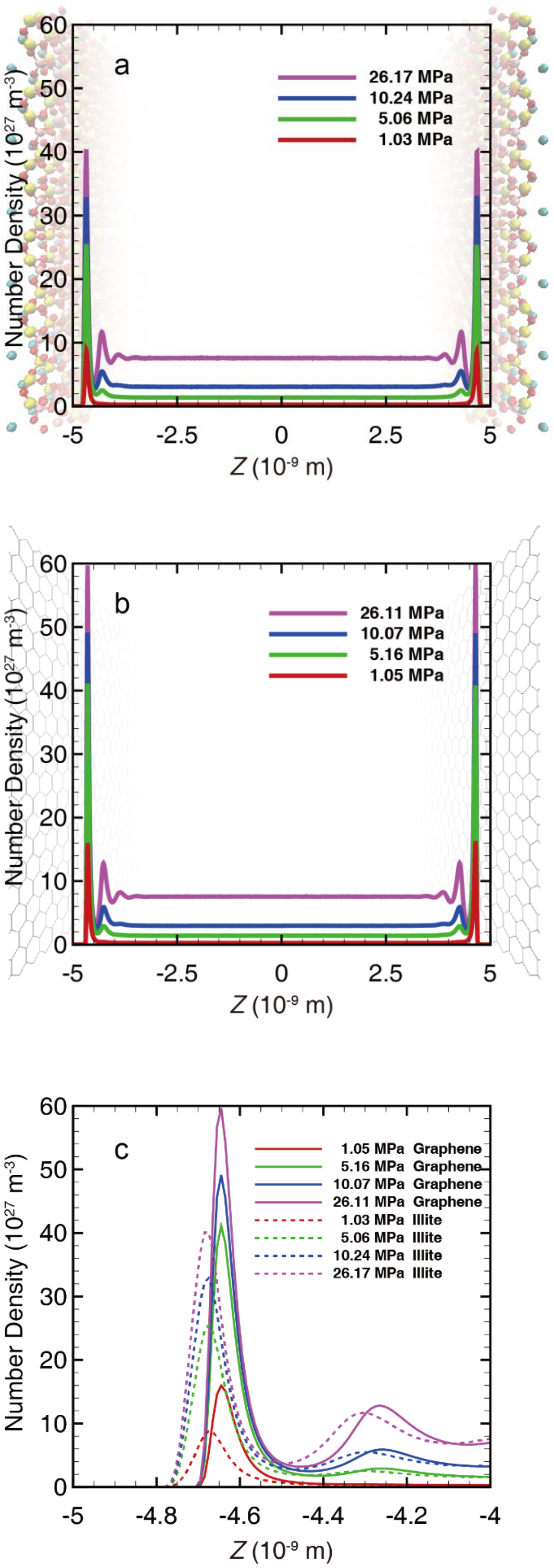

Supplementary Figure S2 | Gas atoms number density distribution. (a) Illite cases; (b) Graphene cases; (c) Density peak near the solid.

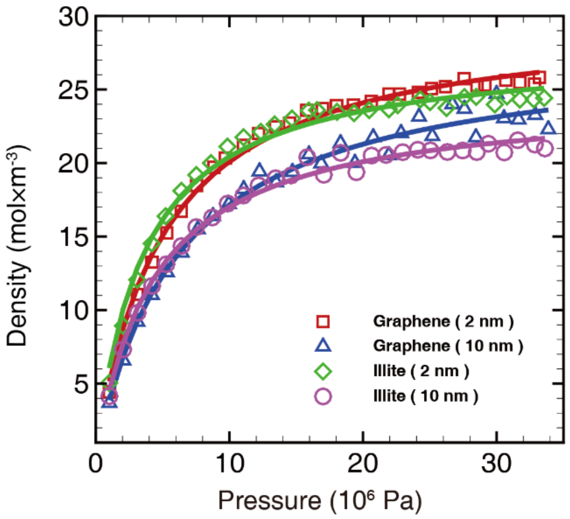

Supplementary Figure S3 | Langmuir isotherms of the adsorption layer. The MD results (symbols) are fitted by Langmuir isotherms (line) as Supplementary Equation (S2).

Supplementary Table S1 | TMAC of illite cases in Fig. 5 of the main text

Case	10 nm<br>Low Pressure	10 nm<br>High Pressure	2 nm<br>Low Pressure	2 nm<br>High Pressure
TMAC_ad	1.00	2.00	1.26	2.00
TMAC_in	1.16	2.00	1.57	1.95
TMAC_BKT	1.32	1.00	1.67	1.95

Supplementary Table S2 | TMAC of graphene cases in Fig. 6 of the main text

Case	10 nm Low Pressure	10 nm High Pressure
TMAC_ad	0.0104	0.0056
TMAC_in	0.8000	0.8700
TMAC_BKT	0.2000	0.0440

Supplementary Table S3 | Parameters of shale gas reservoir for permeability correction model comparison

Parameter	Value
Porosity	5 %
Shale Density	2200 kg m^{-3}
Langmuir Pressure	6.895×10^{6} Pa (1000 psi)
Gas Molar Mass	16.043×10^{-3} kg mol^{-1}

Supplementary Methods

Molecular Dynamic Simulation

The simulations were completed in two different channel (parallel plates) models. The surface area (x and y direction) of the solid wall was 31.164 nm×5.398 nm in the illitic model and 30.012 nm×4.474 nm in the graphene model. The distances between the parallel walls were assigned to be 2 nm and 10 nm in the z-direction for the different cases and were treated as rigid walls. Periodic boundary conditions were imposed in all three directions. Flow direction was in the x-direction. The atom interactions were modeled with the cut and shifted LJ 6-12 model with a radial cutoff at 3σ, where σ corresponds to the distance at which the potential between the two atoms goes to zero, and $\sigma = 0.381$ nm for methane. Lorentz-Berthelot mixing rules were employed, and the parameters were obtained from previous literature[1-3].

The MD simulations were performed with the open-sourced package LAMMPS[4], while the authors programmed some of the new commands. First, the adsorption isotherms were obtained from a combination of grand canonical molecular dynamics (GCMC) and an NVT-MD simulation (see Supplementary Fig. S3). The number density n can be converted to molar concentration c_m as:

$$c_m = n / N_A \tag{S1}$$

where N_A is the Avogadro constant. Because the monolayer adsorption is dominated in these cases, the adsorption isotherms can be described by a Langmuir form:

$$V_{ad}\left(p\right) = V_L \frac{p}{p + P_L} \tag{S2}$$

where V_{ad} is the adsorption amount; p is the gas pressure; V_L is the Langmuir volume; and P_L is the Langmuir pressure, which refers to the pressure at which half of the Langmuir volume

can be adsorbed. The width of the adsorbed region was set to $\gamma\sigma$, and γ is set to 0.8 for methane empirically; then, the density of the adsorbed region ρ_{ad} could be calculated as:

$$\rho_{ad}\left(p\right)=V_L\frac{p}{p+p_L}\frac{\rho_s}{\phi\left(1-r^{*2}\right)V_m} \tag{S3}$$

where ρ_s is the density of shale; ϕ is the porosity; r^* is the radius ratio of the inner region and the entire pore; and V_m is the molar volume.

The atom distribution models under certain pressure conditions were generated via the same method, and imported to the RPM simulations and the EFM simulations. In the RPM simulations, the reflected probability was assigned as 0.005 and 0.01 for different cases. A small density gradient existed along the flow direction, which still fit the linear type and did not affect the macroscopic parameters. In the EFM simulations, the force was assigned as 1×10^{-4} and 5×10^{-3} Kcal mol^{-1} Å^{-1} (1 Kcal mol^{-1} Å^{-1}= 4.184×10^{13} N mol^{-1}) for different cases to obtain reasonable results. The Nose-Hoover thermostat with a time step of 1 fs was imposed to maintain a constant temperature, and the velocity along the x-direction was excluded for temperature calculation. After equilibrating for 10 ns, the simulation was started for another 100 ns for properties accumulating (1000 ns for relatively low-pressure cases, to eliminate noise). The velocity and flow rates were recorded by bins of 0.1 Å×0.1 Å along the x-z plane.

Momentum transport model from kinetic theory

Assuming the Maxwell-Boltzmann speed distribution was valid in this case, molecules crossing a plane of constant z would have travelled an average distance of $\lambda\cos\theta$ since the last collision, where θ is the angle of velocity to the z-axis. Owing to the presence of density oscillation, the total x-momentum flux across the unit area perpendicular to the z-axis in unit time is (the molecules were moving from a faster region to a slower region):

$$
\begin{aligned}
J_{xz} &= \int_0^{\infty}\int_0^{\pi} v\cos\theta f\left(v\right)dv\cdot\frac{1}{2}\sin\theta d\theta\cdot\frac{\partial}{\partial z}\left(nm\bar{u}_x\right)\lambda\cos\theta \\
&= \frac{1}{2}\lambda\int_0^{\infty} vf\left(v\right)dv\left(nm\frac{\partial\bar{u}_x}{\partial z}+\bar{u}_x m\frac{\partial n}{\partial z}\right)\int_0^{\pi}\cos^2\theta\sin\theta d\theta \\
&= \frac{1}{3}\bar{\rho}\lambda\left\langle v\right\rangle\left(\frac{\partial\bar{u}_x}{\partial z}\right)+\frac{1}{3}\bar{u}_x\lambda\left\langle v\right\rangle\frac{\partial\bar{\rho}}{\partial z}
\end{aligned}
\tag{S4}
$$

where $\bar{\rho}=nm$; n is the number density; m is the mass of the particle; λ is the molecular mean free path; and $f\left(v\right)$ is the speed distribution function. The expressions along other directions occur in the same form.

Dual-Region Model

According to the assumption and simplification in this work, the governing equations reduce to:

$$
\begin{cases}
\dfrac{d^2u_{in}}{dz^2}=\dfrac{1}{\eta_{in}}\dfrac{dp}{dx} & \left(z\le z_{ad}\right) \\
\dfrac{d^2u_{ad}}{dz^2}=\dfrac{1}{\eta_{ad}}\dfrac{\rho_{ad}}{\rho_{in}}\dfrac{dp}{dx} & \left(z_{ad}<z\le\dfrac{H}{2}\right)
\end{cases}
\tag{S5}
$$

where z_{ad} is the position of the virtual surface between the adsorbed region and the inner region; H is the effective channel width; η is the viscosity which is corrected with the same method as the BKT model[5,6]; and the subscripts in and ad denote the inner region and the adsorbed region, respectively. Notably, the chemical potential is assumed to be at equilibrium across the channel. The slip boundary conditions at the two surfaces are (in the same form as the BKT model):

$$
\begin{cases}
u_{in}\left(z_{ad}\right)-u_{ad}\left(z_{ad}\right)=\dfrac{2-\sigma_{v,in}}{\sigma_{v,in}}\left[\dfrac{Kn_{in}}{1-bKn_{in}}\left(\dfrac{\partial u}{\partial n}\right)_{in}\right]\dfrac{\alpha-1}{\alpha} \\
u_{ad}\left(\dfrac{H}{2}\right)-u_w=\dfrac{2-\sigma_{v,ad}}{\sigma_{v,ad}}\left[\dfrac{Kn_{ad}}{1-bKn_{ad}}\left(\dfrac{\partial u}{\partial n}\right)_{ad}\right] \\
\left.\dfrac{\partial u}{\partial z}\right|_{z=0}=0 \\
\left.\dfrac{du_{ad}}{dz}\right|_{z=z_{ad}}=\dfrac{1}{\alpha}\left.\dfrac{du_{in}}{dz}\right|_{z=z_{ad}}
\end{cases}
\tag{S6}
$$

where u_w is the wall's velocity ($u_w = 0$ in this case); σ_v is TMAC; Kn is the Knudsen number; b is a slip coefficient and often taken as -1 for channel flow and tube flow; $\left(\frac{\partial u}{\partial n}\right)$ is the normal velocity gradient at the surface; and α is the ratio of density between the adsorbed region and the inner region. Then, the velocity profile can be expressed as equation (6) in the main text, and the expressions for the corresponding coefficients are:

$$\begin{cases} w_1 = -h^* s_{Kn,in} \frac{\alpha - 1}{\alpha} - \frac{1}{4} h^{*2} \\ + \frac{\alpha}{\beta} \left[\frac{h^{*2}}{4} \left(-1 + \frac{2\beta}{\alpha^2} \right) + \left(-s_{Kn,ad} - \frac{1}{4} \right) + h^* \left(s_{Kn,ad} + \frac{1}{2} \right) \left(1 - \frac{\beta}{\alpha^2} \right) \right] \\ w_2 = -h^* \left(1 - \frac{\beta}{\alpha^2} \right) \\ w_3 = \left(-s_{Kn,ad} - \frac{1}{4} \right) + h^* \left(s_{Kn,ad} + \frac{1}{2} \right) \left(1 - \frac{\beta}{\alpha^2} \right) \\ s_{Kn} = \frac{2 - \sigma_v}{\sigma_v} \frac{Kn}{1 - bKn} \end{cases} \tag{S7}$$

According to the expressions of the dual-region model, the model will reduce to the BKT model if no adsorption effect exists, namely, $\alpha = 1$ and $\beta = 1$.

TMAC is sensitive to the gas and solid surface conditions[7], which is adjusted to well describe the velocity profile, and is often taken to be unity for most of the practical engineering conditions. In these cases, TMAC increases as the pressure increases, which indicates that the transport turns to be dominated by the gas–gas interaction gradually, and the fraction of the diffusively reflected gas molecules increases (see Supplementary Table S1 and S2). However, TMAC is always beyond unity, because the Kn is an averaged value for the region with strong density oscillation, and TMAC should be adjusted to get a reasonable s_{Kn} for boundary condition. The values of TMAC in Fig. 7 in the main text were obtained via linear interpolation with the TMACs from MD simulations for various pressure values. On the other hand, the TMAC was assign as 1 in the

permeability correction case, consistent with the previous works.

Permeability Correction Function

The permeability model originates from the capillary tube model[8], therefore, equation (6) in the main text should be converted to the tube model as:

$$\begin{cases} u_{in}(r) = \dfrac{1}{4\eta_{in}} \dfrac{dp}{dx}\left(r^2 + R^2 w_1\right) & \left(r \le r_i\right) \\ u_{ad}(r) = \dfrac{1}{4\eta_{in}} \dfrac{\alpha}{\beta} \dfrac{dp}{dx}\left(r^2 + rRw_2 + R^2 w_3\right) & \left(r_i < r \le R\right) \end{cases} \tag{S8}$$

where r is the position in the tube; R is the effective radius of the tube; r_i is the position of the virtual surface between the adsorbed region and the inner region; and the coefficients are:

$$\begin{cases} w_1 = -2r^* s_{Kn,in} \dfrac{\alpha - 1}{\alpha} - r^{*2} \\ \quad + \dfrac{\alpha}{\beta}\left[r^{*2}\left(-1 + \dfrac{2\beta}{\alpha^2}\right) - \left(2s_{Kn,ad} + 1\right) + 2r^*\left(1 - \dfrac{\beta}{\alpha^2}\right)\left(s_{Kn,ad} + 1\right)\right] \\ w_2 = -2r^*\left(1 - \dfrac{\beta}{\alpha^2}\right) \\ w_3 = -\left(2s_{Kn,ad} + 1\right) + 2r^*\left(1 - \dfrac{\beta}{\alpha^2}\right)\left(s_{Kn,ad} + 1\right) \end{cases} \tag{S9}$$

Integrating the flux across the tube as:

$$Q = \int_0^R 2\pi r \cdot u(r)\rho(r)dr \tag{S10}$$

where

$$\rho(r) = \begin{cases} \rho_{in} & \left(r \le r_i\right) \\ \rho_{ad} & \left(r_i < r \le R\right) \end{cases}$$

On the other hand,

$$Q = -\pi R^2 f k_\infty \frac{\rho_{in}}{\eta_{in}} \frac{dp}{dx} \tag{S11}$$

where k_∞ is the intrinsic permeability which equals $R^2/8$ in the tube flow. Then, the expression of equation (10) in the main text can be obtained.

Supplementary References

1 Firouzi, M. & Wilcox, J. Molecular modeling of carbon dioxide transport and storage in porous carbon-based materials. *Microporous Mesoporous Mater.* **158**, 195–203 (2012).

2 Jin, Z. & Firoozabadi, A. Methane and carbon dioxide adsorption in clay-like slit pores by Monte Carlo simulations. *Fluid Phase Equilib.* **360**, 456–465 (2013).

3 Heinz, H., Koerner, H., Anderson, K. L., Vaia, R. A. & Farmer, B. Force field for mica-type silicates and dynamics of octadecylammonium chains grafted to montmorillonite. *Chem. Mater.* **17**, 5658–5669 (2005).

4 Plimpton, S. Fast parallel algorithms for short-range molecular dynamics. *J. Comput. Phys.* **117**, 1–19 (1995).

5 Beskok, A., Karniadakis, G. E. & Trimmer, W. Rarefaction and compressibility effects in gas microflows. *J. Fluids Eng.* **118**, 448–456 (1996).

6 Beskok, A. & Karniadakis, G. E. Report: a model for flows in channels, pipes, and ducts at micro and nano scales. *Microscale Thermophys. Eng.* **3**, 43–77 (1999).

7 Zhang, W., Meng, G. & Wei, X. A review on slip models for gas microflows. *Microfluid Nanofluid* **13**, 845–882 (2012).

8 Ziarani, A. S. & Aguilera, R. Knudsen’ s permeability correction for tight porous media. *Transport in Porous Media* **91**, 239–260 (2012).
